# (μ_1_-Methano­lato-κ^1^ *O*)-μ_1_-methoxo-κ^1^ *O*-(μ_2_-2-amino-1-methyl-5*H*-imidazol-4-one-κ^2^ *N*:*N*′)-hexa­carbonyl­dirhenium(I)

**DOI:** 10.1107/S1600536812041700

**Published:** 2012-10-13

**Authors:** M. Schutte, H. G. Visser, A. Roodt

**Affiliations:** a Department of Chemistry, University of the Free State, PO Box 339, Bloemfontein, 9301, South Africa

## Abstract

In the title compound, [Re_2_(CH_3_O)_2_(CO)_6_(C_4_H_6_N_3_O)], the two Re^I^ atoms are linked by a methoxo and methanolato bridge, as well as by a creatinine ligand that coordinates in a bidentate fashion. Three *fac*-carbonyl ligands occupy the rest of the slightly distorted octa­hedral geometry around each Re^I^ atom. The bridging methanolato and methoxo ligands are bent out of the Re_2_O_2_ plane by 49.2 (4) and 47.8 (3)° respectively. This is normally associated with a methanolato-bridging-type coordination rather that the more planar methoxo-type bridging. Furthermore, the creatinine bridging molecule is very slightly distorted from the Re_2_N_2_C plane, indicating that the pyrazolo N atom bonded to the Rh^I^ atom is not protonated. Charge balance can thus only be attained if one assumes a positional disorder for the methanolato/methoxo H atom. All attempts to locate disordered protons around these O atoms were unsuccessful. Four hydrogen bonds, one N—H⋯O and three C—H⋯O, are observed in the structure. The mol­ecules pack in a head-to-head and tail-to-tail fashion when viewed along the *c* axis, in alternating columns.

## Related literature

For the synthesis of the starting material, see: Alberto *et al.* (1996 ▶). For similar Re^I^ meth­oxy-bridged structures, see: Franklin *et al.* (2008 ▶); Klausmeyer & Beckles (2006 ▶). For structures of creatinine, see: Bell *et al.* (1995 ▶); du Pré & Mendel (1955 ▶). For structures with creatinine as a monodentate ligand, see: Canty *et al.* (1979 ▶); Mitewa *et al.* (2002 ▶); Matos Beja *et al.* (1991 ▶); Panfil *et al.* (1995 ▶). For a tetra­nuclear Re^I^ complex, see: Schutte *et al.* (2012*a* ▶). For similar Re^I^ structures, see: Schutte *et al.* (2011 ▶, 2012*b* ▶,*c* ▶). 
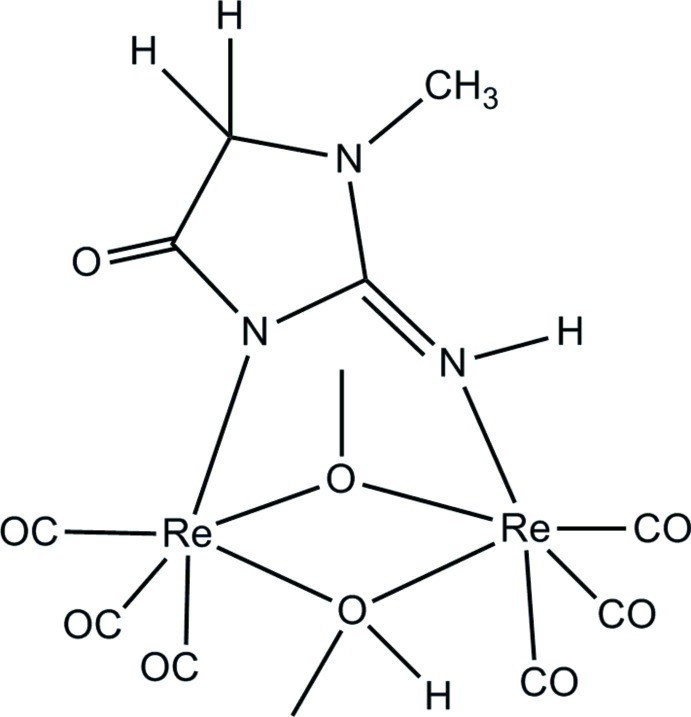


## Experimental

### 

#### Crystal data

[Re_2_(CH_3_O)_2_(CO)_6_(C_4_H_6_N_3_O)]
*M* *_r_* = 714.67
Orthorhombic, 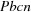

*a* = 24.066 (2) Å
*b* = 10.0715 (8) Å
*c* = 14.5969 (11) Å
*V* = 3538.1 (5) Å^3^
*Z* = 8
Mo *K*α radiation
μ = 13.73 mm^−1^
*T* = 100 K
0.25 × 0.15 × 0.10 mm

#### Data collection

Bruker APEXII CCD diffractometer
Absorption correction: multi-scan (*SADABS*; Bruker, 2008 ▶) *T*_min_ = 0.174, *T*_max_ = 0.371
46753 measured reflections
4276 independent reflections
3920 reflections with *I* > 2σ(*I*)
*R*_int_ = 0.050

#### Refinement

*R*[*F* ^2^ > 2σ(*F* ^2^)] = 0.030
*wR*(*F* ^2^) = 0.078
*S* = 1.16
4269 reflections
236 parameters
H atoms treated by a mixture of independent and constrained refinement
Δρ_max_ = 2.32 e Å^−3^
Δρ_min_ = −2.30 e Å^−3^

### 

Data collection: *APEX2* (Bruker, 2008 ▶); cell refinement: *SAINT-Plus* (Bruker, 2008 ▶); data reduction: *SAINT-Plus*; program(s) used to solve structure: *SHELXS97* (Sheldrick, 2008 ▶); program(s) used to refine structure: *SHELXL97* (Sheldrick, 2008 ▶); molecular graphics: *DIAMOND* (Brandenburg & Putz, 2005 ▶); software used to prepare material for publication: *WinGX* (Farrugia, 1999 ▶).

## Supplementary Material

Crystal structure: contains datablock(s) global, I. DOI: 10.1107/S1600536812041700/tk5155sup1.cif

Structure factors: contains datablock(s) I. DOI: 10.1107/S1600536812041700/tk5155Isup2.hkl

Additional supplementary materials:  crystallographic information; 3D view; checkCIF report

## Figures and Tables

**Table 1:** Selected bond lengths (Å)

Re1—C11	1.886 (6)
Re1—C13	1.908 (7)
Re1—C12	1.918 (8)
Re1—O2	2.149 (4)
Re1—N3	2.150 (5)
Re1—O1	2.153 (4)
Re2—C21	1.849 (5)
Re2—C22	1.935 (6)
Re2—C23	1.949 (6)
Re2—O2	2.065 (4)
Re2—O1	2.073 (4)
Re2—N1	2.136 (5)

**Table 2:** Hydrogen-bond geometry (Å, °)

*D*—H⋯*A*	*D*—H	H⋯*A*	*D*⋯*A*	*D*—H⋯*A*
N3—H3⋯O31^i^	0.92 (8)	2.17 (8)	3.061 (6)	162 (7)
C2—H2*B*⋯O13^ii^	0.96	2.54	3.453 (9)	159
C2—H2*C*⋯O23^ii^	0.96	2.61	3.504 (9)	154
C34—H34*A*⋯O31^i^	0.96	2.44	3.332 (7)	155

Symmetry codes: (i) 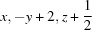; (ii) 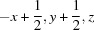.

**Fig. 1.**
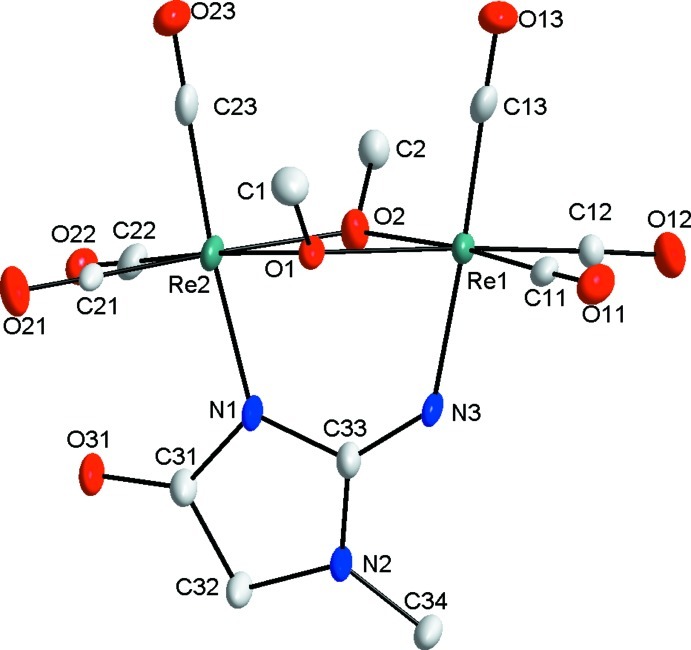
Representation of the title compound, showing the numbering scheme and displacement ellipsoids (50% probability).

**Fig. 2.**
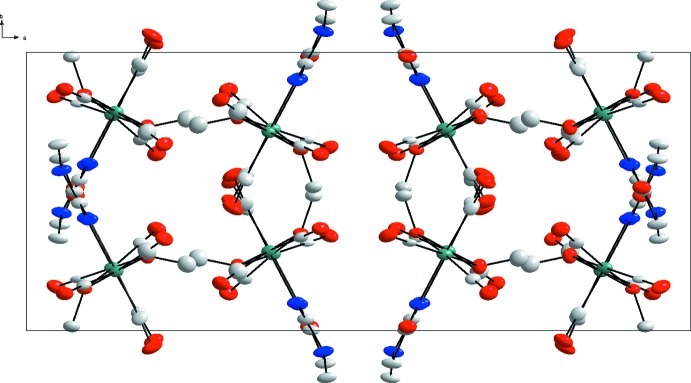
Packing and hydrogen interactions (dashed lines) of the title compound in the unit cell. Hydrogen atoms omitted for clarity.

## supplementary crystallographic information

### Comment

C_12_H_12_N_3_O_9_Re_2_ crystallized in the orthorhombic space group *Pbcn* with one unit in the asymmetric unit. The rhenium centres are linked with two methoxy bridges and a creatinine ligand (in a N,N'-bidentate fashion). Three *facial* tricarbonyl ligands occupy the other three positions on the distorted octahedron, Fig. 1 and Table 1. The Re—O—Re (104.01 (18)° and 104.4 (2)°) and O—Re—O (73.71 (16)° and 77.16 (17)°) bond angles compare well to the structure by Klausmeyer & Beckles (2006) that reported 103.66 (1)° and 103.33 (1)° (Re—O—Re) and 76.66 (1)° and 76.21 (1)° (O—Re—O), and to the structure by Franklin *et al.* (2008) that reported 108.24 (1)° and 108.24 (2)° (Re—O—Re) and 71.76 (12)° (O—Re—O) respectively. Creatinine is coordinated to various metal centres in a monodentate fashion (Canty *et al.* 1979, Mitewa *et al.* 2002, Matos Beja *et al.* 1991, Panfil *et al.* 1995) but no structure reports are found where creatinine is coordinated to a metal centre in a bidentate fashion. All bond distances and angles of creatinine in this structure compare well with that of the reported structures of the free ligand with the N—C—N angle the only exception with 121° (Bell *et al.*, 1995) and 120° (du Pré & Mendel, 1955) reported for the free ligand and 123.7 (5)° for the coordinated ligand, respectively. The O—Re—O (73.71 (16)° and 77.16 (17)°) and Re—O—Re (104.01 (18)° and 104.4 (2)°) bond angles compare well to the tetranuclear rhenium(I) cubane-like molecule reported by Schutte *et al.* (2012*a*) with O—Re—O angles that vary between 73.5 (2)° and 75.0 (7)° and Re—O—Re angles that vary between 102.6 (3)° and 104.7 (2)°. This ligand, creatinine, forms part of an ongoing study where different N,N' bidentate and N,N',N'' tridentate ligands are synthesized and coordinated to the rhenium(I) metal centre (Schutte *et al.*, 2011, 2012*b*, 2012*c*). Four intermolecular hydrogen bonds are observed in the structure, Table 2, *i.e*. one N—H···O and three C—H···O. When viewed along the *c* axis, the molecules pack in column-like structures in an alternating head-to-head and tail-to-tail fashion (Fig. 2).

### Experimental

[NEt_4_]_2_[Re(CO)_3_Br_3_] (500 mg, 0.650 mmol), as prepared by Alberto *et al.* (1996), was dissolved in 10 ml of water at pH 2. The pH was increased to pH 6 and after a slight colour change, creatinine (37 mg, 0.325 mmol) was added to the mixture and stirred for 6 h at room temperature. The yellow cuboidal crystals were obtained from the filtrate of the solution.

### Refinement

The methyl and methene H atoms were placed in geometrically idealized positions and constrained to ride on its parent atoms with *U*_iso_(H) = 1.5*U*_eq_(C) and *U*_iso_(H) = 1.2*U*_eq_(C) and at a distance of 0.96 Å and 0.97 Å respectively. The N-bound H atom was refined freely. A number of reflections were omitted from the final cycles of refinement owing to poor agreement. All attempts to locate disordered protons around O1 and O2 were unsuccessful.

### Crystal data

**Table:**

[Re_2_(CH_3_O)_2_(CO)_6_(C_4_H_6_N_3_O)]	*F*(000) = 2616
*M**_r_* = 714.67	*D*_x_ = 2.683 Mg m^−^^3^
Orthorhombic, *P**b**c**n*	Mo *K*α radiation, λ = 0.71073 Å
Hall symbol: -P 2n 2ab	Cell parameters from 9082 reflections
*a* = 24.066 (2) Å	θ = 3.0–28.3°
*b* = 10.0715 (8) Å	µ = 13.73 mm^−^^1^
*c* = 14.5969 (11) Å	*T* = 100 K
*V* = 3538.1 (5) Å^3^	Cuboid, yellow
*Z* = 8	0.25 × 0.15 × 0.10 mm

### Data collection

**Table:**

Bruker APEXII CCD diffractometer	3920 reflections with *I* > 2σ(*I*)
Graphite monochromator	*R*_int_ = 0.050
φ and ω scans	θ_max_ = 28°, θ_min_ = 2.8°
Absorption correction: multi-scan (*SADABS*; Bruker, 2008)	*h* = −31→31
*T*_min_ = 0.174, *T*_max_ = 0.371	*k* = −13→12
46753 measured reflections	*l* = −19→18
4276 independent reflections	

### Refinement

**Table:**

Refinement on *F*^2^	Primary atom site location: structure-invariant direct methods
Least-squares matrix: full	Secondary atom site location: difference Fourier map
*R*[*F*^2^ > 2σ(*F*^2^)] = 0.03	Hydrogen site location: inferred from neighbouring sites
*wR*(*F*^2^) = 0.078	H atoms treated by a mixture of independent and constrained refinement
*S* = 1.16	*w* = 1/[σ^2^(*F*_o_^2^) + (0.0246*P*)^2^ + 32.0137*P*] where *P* = (*F*_o_^2^ + 2*F*_c_^2^)/3
4269 reflections	(Δ/σ)_max_ = 0.006
236 parameters	Δρ_max_ = 2.32 e Å^−^^3^
0 restraints	Δρ_min_ = −2.30 e Å^−^^3^

### Special details

**Table:**

Geometry. All s.u.'s (except the s.u. in the dihedral angle between two l.s. planes) are estimated using the full covariance matrix. The cell s.u.'s are taken into account individually in the estimation of s.u.'s in distances, angles and torsion angles; correlations between s.u.'s in cell parameters are only used when they are defined by crystal symmetry. An approximate (isotropic) treatment of cell s.u.'s is used for estimating s.u.'s involving l.s. planes.
Refinement. Refinement of *F*^2^ against ALL reflections. The weighted *R*-factor *wR* and goodness of fit *S* are based on *F*^2^, conventional *R*-factors *R* are based on *F*, with *F* set to zero for negative *F*^2^. The threshold expression of *F*^2^ > 2σ(*F*^2^) is used only for calculating *R*-factors(gt) *etc*. and is not relevant to the choice of reflections for refinement. *R*-factors based on *F*^2^ are statistically about twice as large as those based on *F*, and *R*- factors based on ALL data will be even larger.

### Fractional atomic coordinates and isotropic or equivalent isotropic displacement parameters (Å^2^)

**Table:**

	*x*	*y*	*z*	*U*_iso_*/*U*_eq_	
Re1	0.364485 (10)	0.71291 (2)	0.050452 (15)	0.01847 (7)	
Re2	0.370469 (11)	0.72576 (2)	−0.177323 (15)	0.02132 (8)	
O21	0.4552 (2)	0.6573 (5)	−0.3213 (3)	0.0324 (11)	
O22	0.3040 (2)	0.8637 (5)	−0.3303 (3)	0.0358 (11)	
O12	0.2927 (2)	0.8300 (5)	0.2038 (3)	0.0315 (10)	
C21	0.4229 (2)	0.6840 (5)	−0.2654 (3)	0.0164 (10)	
C12	0.3184 (4)	0.7864 (8)	0.1449 (5)	0.0397 (12)	
C22	0.3280 (3)	0.8139 (6)	−0.2720 (4)	0.0225 (12)	
O13	0.3108 (2)	0.4401 (5)	0.0824 (3)	0.0387 (12)	
O23	0.3083 (2)	0.4642 (5)	−0.2175 (3)	0.0348 (11)	
C13	0.3295 (3)	0.5449 (7)	0.0687 (4)	0.0265 (13)	
C23	0.3310 (3)	0.5609 (6)	−0.2029 (4)	0.0229 (12)	
O1	0.41283 (18)	0.6491 (4)	−0.0656 (2)	0.0201 (8)	
O2	0.3181 (2)	0.7690 (5)	−0.0693 (3)	0.0262 (10)	
C1	0.4321 (3)	0.5153 (6)	−0.0685 (4)	0.0280 (13)	
H1C	0.401	0.4564	−0.0754	0.042*	
H1A	0.4514	0.495	−0.0126	0.042*	
H1B	0.457	0.5045	−0.1194	0.042*	
C11	0.4133 (3)	0.6643 (5)	0.1458 (4)	0.0203 (11)	
O11	0.4425 (2)	0.6333 (4)	0.2055 (3)	0.0294 (10)	
O31	0.4263 (2)	0.9857 (4)	−0.2855 (3)	0.0277 (10)	
N1	0.4107 (2)	0.9064 (5)	−0.1391 (3)	0.0230 (11)	
N3	0.4054 (2)	0.8987 (5)	0.0237 (3)	0.0231 (11)	
N2	0.4472 (3)	1.0758 (5)	−0.0571 (3)	0.0288 (13)	
C33	0.4202 (3)	0.9583 (6)	−0.0515 (4)	0.0227 (12)	
C31	0.4282 (3)	0.9978 (6)	−0.2020 (4)	0.0243 (12)	
C34	0.4523 (3)	1.1691 (6)	0.0173 (4)	0.0285 (14)	
H34C	0.4193	1.2223	0.0208	0.043*	
H34B	0.4839	1.2254	0.007	0.043*	
H34A	0.4571	1.1216	0.0738	0.043*	
C32	0.4501 (3)	1.1178 (6)	−0.1519 (4)	0.0287 (14)	
H32A	0.488	1.1377	−0.1699	0.034*	
H32B	0.427	1.1951	−0.1628	0.034*	
C2	0.2586 (4)	0.7391 (8)	−0.0714 (5)	0.0397 (12)	
H2B	0.2411	0.7755	−0.0179	0.06*	
H2A	0.2534	0.6446	−0.0724	0.06*	
H2C	0.2423	0.7775	−0.1253	0.06*	
H3	0.418 (3)	0.944 (8)	0.075 (5)	0.03 (2)*	

### Atomic displacement parameters (Å^2^)

**Table:**

	*U*^11^	*U*^22^	*U*^33^	*U*^12^	*U*^13^	*U*^23^
Re1	0.02881 (14)	0.01810 (12)	0.00849 (11)	−0.00294 (9)	0.00026 (8)	0.00018 (7)
Re2	0.03762 (15)	0.01828 (12)	0.00805 (11)	−0.00136 (9)	−0.00146 (8)	−0.00093 (7)
O21	0.052 (3)	0.028 (2)	0.018 (2)	0.010 (2)	0.0100 (19)	0.0049 (18)
O22	0.050 (3)	0.032 (3)	0.025 (2)	0.004 (2)	−0.011 (2)	0.004 (2)
O12	0.044 (3)	0.028 (2)	0.022 (2)	0.005 (2)	0.0111 (19)	0.0002 (19)
C21	0.031 (3)	0.008 (2)	0.009 (2)	−0.001 (2)	−0.002 (2)	0.0019 (18)
C12	0.050 (3)	0.041 (3)	0.028 (3)	0.004 (2)	0.001 (2)	0.002 (2)
C22	0.034 (3)	0.018 (3)	0.015 (3)	0.002 (2)	−0.003 (2)	−0.004 (2)
O13	0.060 (3)	0.028 (2)	0.028 (2)	−0.020 (2)	0.004 (2)	−0.002 (2)
O23	0.053 (3)	0.028 (2)	0.023 (2)	−0.013 (2)	−0.003 (2)	−0.0039 (19)
C13	0.037 (4)	0.029 (3)	0.013 (3)	−0.005 (3)	−0.001 (2)	−0.006 (2)
C23	0.033 (3)	0.027 (3)	0.009 (2)	0.003 (3)	0.001 (2)	0.000 (2)
O1	0.034 (2)	0.0147 (18)	0.0113 (17)	0.0002 (17)	0.0006 (15)	0.0012 (14)
O2	0.031 (2)	0.034 (2)	0.014 (2)	0.0017 (19)	0.0014 (16)	−0.0003 (17)
C1	0.040 (4)	0.019 (3)	0.025 (3)	0.002 (3)	0.001 (3)	0.003 (2)
C11	0.032 (3)	0.015 (3)	0.014 (2)	−0.004 (2)	0.000 (2)	−0.003 (2)
O11	0.043 (3)	0.024 (2)	0.022 (2)	0.0000 (19)	−0.0074 (19)	0.0003 (17)
O31	0.049 (3)	0.024 (2)	0.0098 (18)	0.001 (2)	0.0006 (18)	0.0023 (16)
N1	0.044 (3)	0.018 (2)	0.007 (2)	−0.003 (2)	0.0014 (19)	−0.0002 (17)
N3	0.044 (3)	0.018 (2)	0.007 (2)	−0.004 (2)	−0.0002 (19)	−0.0021 (17)
N2	0.059 (4)	0.018 (2)	0.009 (2)	−0.008 (2)	0.006 (2)	−0.0010 (18)
C33	0.039 (3)	0.018 (3)	0.011 (2)	0.000 (2)	0.003 (2)	0.000 (2)
C31	0.044 (4)	0.015 (3)	0.013 (2)	0.002 (2)	0.003 (2)	0.002 (2)
C34	0.055 (4)	0.016 (3)	0.014 (3)	−0.006 (3)	0.002 (3)	−0.002 (2)
C32	0.060 (5)	0.015 (3)	0.011 (3)	−0.005 (3)	0.002 (3)	0.002 (2)
C2	0.050 (3)	0.041 (3)	0.028 (3)	0.004 (2)	0.001 (2)	0.002 (2)

### Geometric parameters (Å, º)

**Table:**

Re1—C11	1.886 (6)	C1—H1A	0.96
Re1—C13	1.908 (7)	C1—H1B	0.96
Re1—C12	1.918 (8)	C11—O11	1.161 (7)
Re1—O2	2.149 (4)	O31—C31	1.225 (7)
Re1—N3	2.150 (5)	N1—C31	1.367 (7)
Re1—O1	2.153 (4)	N1—C33	1.399 (7)
Re2—C21	1.849 (5)	N3—C33	1.300 (7)
Re2—C22	1.935 (6)	N3—H3	0.92 (8)
Re2—C23	1.949 (6)	N2—C33	1.353 (8)
Re2—O2	2.065 (4)	N2—C34	1.442 (7)
Re2—O1	2.073 (4)	N2—C32	1.449 (7)
Re2—N1	2.136 (5)	C31—C32	1.507 (8)
O21—C21	1.159 (7)	C34—H34C	0.96
O22—C22	1.143 (7)	C34—H34B	0.96
O12—C12	1.147 (9)	C34—H34A	0.96
O13—C13	1.165 (8)	C32—H32A	0.97
O23—C23	1.137 (8)	C32—H32B	0.97
O1—C1	1.426 (7)	C2—H2B	0.96
O2—C2	1.464 (10)	C2—H2A	0.96
C1—H1C	0.96	C2—H2C	0.96
			
C11—Re1—C13	86.7 (3)	C2—O2—Re1	118.1 (4)
C11—Re1—C12	86.0 (3)	Re2—O2—Re1	104.4 (2)
C13—Re1—C12	89.2 (3)	O1—C1—H1C	109.5
C11—Re1—O2	172.7 (2)	O1—C1—H1A	109.5
C13—Re1—O2	96.8 (2)	H1C—C1—H1A	109.5
C12—Re1—O2	100.5 (3)	O1—C1—H1B	109.5
C11—Re1—N3	94.3 (2)	H1C—C1—H1B	109.5
C13—Re1—N3	177.2 (2)	H1A—C1—H1B	109.5
C12—Re1—N3	93.4 (3)	O11—C11—Re1	178.7 (5)
O2—Re1—N3	82.02 (18)	C31—N1—C33	108.2 (5)
C11—Re1—O1	99.6 (2)	C31—N1—Re2	122.5 (4)
C13—Re1—O1	94.8 (2)	C33—N1—Re2	129.1 (4)
C12—Re1—O1	173.3 (2)	C33—N3—Re1	132.9 (4)
O2—Re1—O1	73.71 (16)	C33—N3—H3	111 (5)
N3—Re1—O1	82.47 (17)	Re1—N3—H3	115 (5)
C21—Re2—C22	88.2 (2)	C33—N2—C34	124.4 (5)
C21—Re2—C23	90.4 (2)	C33—N2—C32	109.7 (5)
C22—Re2—C23	89.8 (2)	C34—N2—C32	121.7 (5)
C21—Re2—O2	174.2 (2)	N3—C33—N2	125.9 (5)
C22—Re2—O2	97.3 (2)	N3—C33—N1	123.7 (5)
C23—Re2—O2	91.6 (2)	N2—C33—N1	110.5 (5)
C21—Re2—O1	97.3 (2)	O31—C31—N1	126.1 (6)
C22—Re2—O1	173.2 (2)	O31—C31—C32	125.1 (5)
C23—Re2—O1	94.2 (2)	N1—C31—C32	108.7 (5)
O2—Re2—O1	77.16 (17)	N2—C34—H34C	109.5
C21—Re2—N1	93.8 (2)	N2—C34—H34B	109.5
C22—Re2—N1	92.0 (2)	H34C—C34—H34B	109.5
C23—Re2—N1	175.5 (2)	N2—C34—H34A	109.5
O2—Re2—N1	84.11 (19)	H34C—C34—H34A	109.5
O1—Re2—N1	83.59 (17)	H34B—C34—H34A	109.5
O21—C21—Re2	179.1 (5)	N2—C32—C31	102.3 (5)
O12—C12—Re1	177.1 (7)	N2—C32—H32A	111.3
O22—C22—Re2	177.5 (6)	C31—C32—H32A	111.3
O13—C13—Re1	176.2 (6)	N2—C32—H32B	111.3
O23—C23—Re2	179.4 (6)	C31—C32—H32B	111.3
C1—O1—Re2	119.3 (3)	H32A—C32—H32B	109.2
C1—O1—Re1	118.8 (3)	O2—C2—H2B	109.5
Re2—O1—Re1	104.01 (18)	O2—C2—H2A	109.5
C2—O2—Re2	122.5 (4)	O2—C2—H2C	109.5

### Hydrogen-bond geometry (Å, º)

**Table:**

*D*—H···*A*	*D*—H	H···*A*	*D*···*A*	*D*—H···*A*
N3—H3···O31^i^	0.92 (8)	2.17 (8)	3.061 (6)	162 (7)
C2—H2*B*···O13^ii^	0.96	2.54	3.453 (9)	159
C2—H2*C*···O23^ii^	0.96	2.61	3.504 (9)	154
C34—H34*A*···O31^i^	0.96	2.44	3.332 (7)	155

Symmetry codes: (i) *x*, −*y*+2, *z*+1/2; (ii) −*x*+1/2, *y*+1/2, *z*.
